# *Xist* RNA in action: Past, present, and future

**DOI:** 10.1371/journal.pgen.1008333

**Published:** 2019-09-19

**Authors:** Agnese Loda, Edith Heard

**Affiliations:** 1 Directors’ research, European Molecular Biology Laboratory (EMBL), Heidelberg, Germany; 2 Collège de France, Paris, France; University of Pennsylvania, UNITED STATES

## Abstract

In mammals, dosage compensation of sex chromosomal genes between females (XX) and males (XY) is achieved through X-chromosome inactivation (XCI). The X-linked X-inactive-specific transcript (*Xist*) long noncoding RNA is indispensable for XCI and initiates the process early during development by spreading *in cis* across the X chromosome from which it is transcribed. During XCI, *Xist* RNA triggers gene silencing, recruits a plethora of chromatin modifying factors, and drives a major structural reorganization of the X chromosome. Here, we review our knowledge of the multitude of epigenetic events orchestrated by *Xist* RNA to allow female mammals to survive through embryonic development by establishing and maintaining proper dosage compensation. In particular, we focus on recent studies characterizing the interaction partners of *Xist* RNA, and we discuss how they have affected the field by addressing long-standing controversies or by giving rise to new research perspectives that are currently being explored. This review is dedicated to the memory of Denise Barlow, pioneer of genomic imprinting and functional long noncoding RNAs (lncRNAs), whose work has revolutionized the epigenetics field and continues to inspire generations of scientists.

## Introduction

X-inactive–specific transcript (*Xist*) RNA was one of the first long noncoding RNAs (lncRNAs) to be discovered in the early 1990s [1–5], a decade before the Human Genome Project (HGP) revealed that the large majority of our genome accounts for noncoding sequences [6]. Two other famous lncRNAs were discovered around that time: *H19*, at the *Igf2* imprinted cluster [7,8], and *Airn*, antisense to the imprinted *Igf2r* gene. *Airn* was uncovered by Denise Barlow as the first example of lncRNA playing a direct role in controlling the imprinted expression of neighboring genes [9,10]. As all three lncRNAs were implicated in epigenetic processes, their discovery raised expectations that epigenetic regulation might be a common feature of lncRNAs. Since then, thousands of intergenic, intronic, and antisense lncRNAs have been identified [11–14], and their widespread transcriptional activity has been increasingly recognized not only in humans but in many different organisms including mouse, zebrafish, and yeast [15–18]. Although their identification has become easier, defining the biological relevance of lncRNAs has remained challenging. Indeed, like *Airn*, it took nearly 30 years of research to characterize the function of *Xist*, and several important questions still need to be answered.

## The X-inactivation centre: A paradigm for the study of lncRNAs

**Fig 1:**
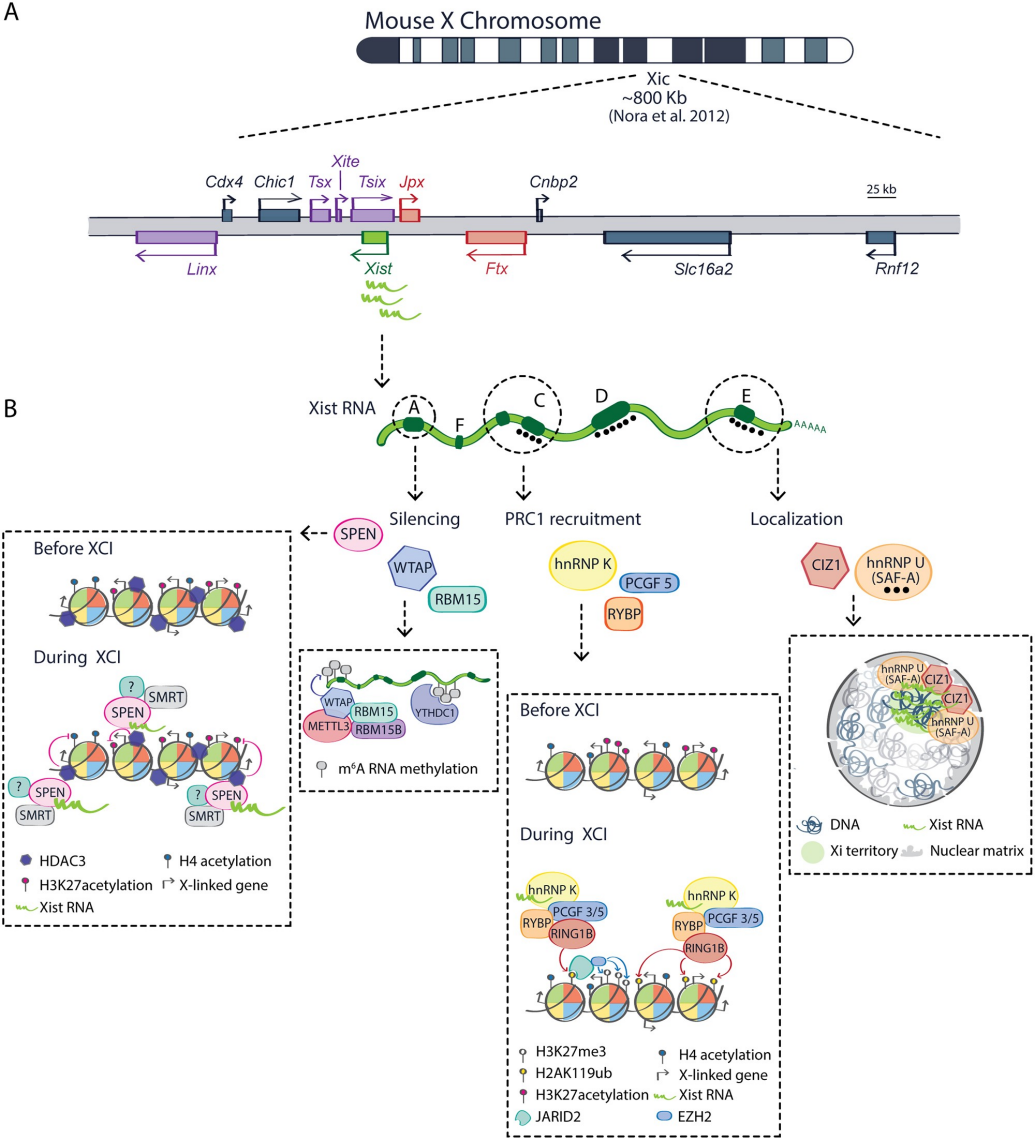
*Xist*. A multi-tasking X-linked transcript. (A) Genomic organization of the mouse X-inactivation centre (Xic) and its location along the X chromosome (adapted from[134]). *Xist* regulators within the Xic are shown: lncRNAs acting as positive regulators in red, negative regulators in violet. *Rnf12*, a protein coding gene activating *Xist in trans*, is also shown. (B) Schematic representation of *Xist* RNA showing its tandem repeats. Factors identified as *Xist* RNA interactors are shown below repeats A, B, C, and E. See main text for details. Xic, X-inactivation center.

*Xist* RNA is the master regulator of X-chromosome inactivation (XCI), the epigenetic process that equalizes the dosage of X-linked genes between female (XX) and male (XY) mammals. At the onset of XCI, *Xist* is up-regulated from one of the two X chromosomes, and its RNA spreads *in cis* along the entire X and triggers the inactivation of most of its >1,000 genes. Ultimately, *Xist* spreading results in the conversion of one of the two active X chromosomes into a uniquely organized heterochromatic entity known as the “Barr body” [19]. Although XCI is a chromosome-wide process, some X-linked genes escape silencing and remain expressed from both the active (Xa) and the inactive (Xi) chromosome. This concerns 12%–20% of human X-linked genes and 3%–7% in mouse and may play an important role in female development and disease susceptibility [20]. For example, only 1% of human female embryos carrying a single X (XO) survive to term, and all of them are affected by Turner syndrome [21,22]. On the other hand, XX women are more susceptible than XY men to autoimmune diseases, and women with supernumerary X chromosomes (XXX) are even more susceptible [23,24]. As the up-regulation of both *Xist* alleles would lead to silencing of both X chromosomes and presumably consequent cell death, the spatio-temporal expression of *Xist* during development needs to be accurately regulated. Historically, the X-linked minimal genetic region that is necessary and sufficient to initiate XCI in female cells has been defined as the X-inactivation centre (Xic) (Fig 1). The Xic guarantees the monoallelic expression of *Xist* when it is present in two copies. Although the exact extent of the functional Xic is still to be fully determined, it encompasses several lncRNAs that act as regulators of *Xist*. For example, one major repressor of *Xist* in the mouse is *Tsix*, a lncRNA that completely overlaps with the *Xist* transcriptional unit [25]. *Tsix* transcription through the promoter of *Xist* works as a break for *Xist* expression, and accordingly, deleting *Tsix* or terminating its transcription prematurely results in nonrandom inactivation of the mutated X chromosome [26–32]. Other loci that produce lncRNAs at the Xic are *Xite*, *Tsx*, *Linx*, *Jpx*, and *Ftx*, all of which have been proposed to work as either negative or positive regulators of *Xist* [33–40]. Thus, throughout the years, unraveling the mechanisms that orchestrate appropriate *Xist* regulation within the Xic has represented a powerful system to more generally understand how lncRNAs contribute to gene regulation, providing meaningful insights into the roles of antisense transcription and RNA-mediated silencing. This has been covered in several reviews [41–44]. Here we focus on the function of *Xist* RNA itself, from its ability to trigger the formation of facultative heterochromatin by recruiting a multitude of different factors to its impact on nuclear localization and 3D chromosome architecture.

## *Xist* is a multi-tasking RNA molecule essential for XCI

*Xist* was initially proven to be indispensable for XCI by targeted mutagenesis and transgenesis in mouse embryonic stem cells (ESCs) and in mice. Deletion of the *Xist* gene results in skewed inactivation of the wild type X chromosome, indicating that this locus is essential for gene silencing [45,46]. Subsequently, the generation of inducible *Xist* transgenes integrated on autosomes demonstrated that *Xist* RNA alone is sufficient to trigger gene silencing [47–49], although the efficiency of autosomal silencing is lower when compared with the inactivation of X-linked genes [50]. The early transgenic studies also unveiled two key features of *Xist*’s function. First, the ability of *Xist* RNA to trigger gene silencing is strictly dependent on the developmental context. If *Xist* is ectopically induced outside of a critical temporal window during early ESCs differentiation, it will be no longer capable of initiating gene silencing [47]. Second, *Xist* has different tasks, such as cis-localization to the chromosome from which it is expressed and the ability to trigger gene silencing, and these tasks are mediated by genetically independent domains of the RNA [51]. The various functional domains of *Xist* have now been defined by genetic and biochemical approaches and overlap at least partially with blocks of tandem repeats named A–F repeats, which are conserved between human and mouse and were therefore suspected to play a role in the function of *Xist* [2,5,51,52] (Fig 1). By deleting each of these repeat regions or a combination of them in mouse *Xist*-inducible transgenes, the highly conserved repeat A element, consisting of 7.5 copies of a 26 nt core sequence at the 5’ end of *Xist* RNA, was characterized as the necessary element to trigger gene silencing [51]. Intriguingly, deletion of the A-repeat region at the endogenous *Xist* gene seems to affect *Xist* RNA’s capacity to be expressed from its endogenous promoter [53], suggesting that this region maybe important both for regulating transcription and/or processing of *Xist* and for mediating its silencing function. On the other hand, *Xist* RNA localization to the X chromosome appeared to be mediated by a combination of different domains, including repeats C, E, and F, via redundant mechanisms [51,54–61]. Finally, Repeats B and C have been found to be required for the recruitment of the Polycomb repressive complex 1 (PRC1) to the inactive X chromosome (Xi), and PRC1 activity has been proposed to subsequently recruit PRC2 (see the section “hnRNP K”) [60,62–64]. Several studies aimed to characterize putative RNA-binding proteins that *Xist* might recruit to fulfil its multiple and genetically uncoupled functions. In this context, the transcription factor YY1 was reported to mediate *Xist* localization by bridging the repeat C region of the RNA with a “nucleation centre” at the repeat F of the DNA [59]. The heterogeneous nuclear ribonucleoprotein U (hnRNP U, also known as SAF-A) was also shown to interact with *Xist* RNA by binding to regions previously reported to mediate *Xist* localization, including repeat C and a region downstream of repeat E within exon 7 [56,61]. Finally, a 1.2-kb transcript derived from the *Xist* locus known as Rep A and overlapping with *Xist* repeat A, was previously reported to recruit PRC2 to the Xi by directly binding its catalytic subunit: EZH2 [65]. EZH2 activity is responsible for the deposition of H3K27me3, one of the silent chromatin marks acquired along the Xi during XCI [66–71]. Similarly, the chromatin remodeller ATRX was reported to bind to Rep A RNA to reinforce PRC2 recruitment [72].

A major breakthrough in the characterization of the interactome of *Xist* was achieved when three independent proteomic approaches purified *Xist* RNA and its binding proteins in mouse ESCs and fully differentiated cells [73–75]. Amongst the factors that were previously reported to bind *Xist* RNA, only hnRNP U was confirmed by all proteomic studies. On the contrary, neither EZH2 nor any other PRC2 components were identified, but PRC1 proteins were. YY1 was also not pulled down by *Xist* RNA, whereas ATRX was only identified as a weak interactor of *Xist* in one of the three studies [75].

In parallel, the functional relevance of several of the newly identified interactors including SPEN, RBM15, and WTAP was confirmed by two genetic screens aiming to define the key factors accounting for the ability of *Xist* to trigger gene silencing [76,77]. Altogether, these unbiased approaches provided the field with an unprecedented opportunity to dissect the different functions of *Xist* and to identify its key protein partners. The research to understand their mechanisms of action in XCI is currently ongoing and will greatly enhance our general understanding of facultative heterochromatin formation, nuclear localization, and chromosome 3D spatial organization beyond the XCI field.

## Interactors of *Xist* contributing to chromosome-wide gene silencing

### SPEN

The only binding factor of *Xist* RNA that was identified by all proteomic and genetic approaches is SPEN (also known as SHARP, SMRT/HDAC1-associated repressor protein), a 400-kDa protein harbouring four N-terminal RNA recognition motifs (RRMs) and a highly conserved C-terminal Spen paralog and ortholog domain (SPOC). The functional role of SPEN in XCI was initially confirmed by knockdown and knockout experiments in which loss of SPEN resulted in defective silencing of the few X-linked genes that were tested [73,74,76,77]. More recently, the requirement of SPEN to achieve effective XCI was further proven by chromosome-wide analysis of X-linked expression upon differentiation of SPEN null ESCs [78]. In other contexts, SPEN’s SPOC domain is able to recruit SMRT, a component of the transcriptional corepressor complex that activates histone deacetylases and leads to transcriptional repression [79–82]. As the functional depletion of SMRT and HDAC3 in male ESCs carrying an inducible *Xist* allele leads to impaired gene silencing, resembling what is observed after SPEN knockdown, a model was put forward according to which SPEN might initiate transcriptional silencing by actively recruiting HDAC3 via SMRT binding [74]. Indeed, in a recent study exploring the choreography of chromatin changes associated with XCI, histone deacetylation was found to be one of the earliest chromatin alterations induced by *Xist* RNA coating, and HDAC3 activity was specifically shown to promote silencing of most X-linked genes [83]. Nevertheless, HDAC3 does not appear to be robustly recruited to the Xi at the onset of XCI, nor does it seem to be a direct partner of *Xist* RNA, indicating that *Xist* RNA might initiate gene silencing through its interaction with SPEN by activating the prebound HDAC3 rather than by recruiting it de novo along the future Xi [83].

A key indication of SPEN’s function in XCI was the demonstration that it binds to *Xist* RNA via the A repeat [73], originally identified as the functional domain required for gene silencing [51]. Indeed, depletion of SPEN only affects gene silencing without compromising the ability of *Xist* to localize to the Xi *in cis* [77]. However, global hypoacetylation of histone H4 along the Xi is not affected in SPEN knockout ESCs [77], and loss of HDAC3 does not fully prevent XCI to occur but rather delays the process [83], suggesting that other redundant mechanisms are likely to have evolved to guarantee proper XCI, at least during mouse development. Furthermore, as SPEN depletion results in reduced accumulation of the Polycomb-dependent chromatin marks H3K27me3 and H2AK119ub along the Xi, SPEN was suggested to also play a role in Polycomb recruitment [74,77]. However, *Xist* RNA lacking the A repeat is still able to recruit both PRC1 and PRC2 and to create a repressive compartment from which most hallmarks of active transcription, including RNA Pol II and general transcription factors, are excluded [83–86]. Accordingly, three subunits of the PRC1 complex, RING1B, RYBP, and PCGF5, were pulled down by *Xist* RNA lacking the A repeat [73], suggesting that PRC1 is recruited to the Xi independently of the silencing domain of *Xist*. Interestingly, none of the core PRC2 components were pulled down by the full length *Xist* RNA [73–75]. The relative timing of H3K27me3 and H2AK119Ub deposition and their dependency on the A repeat region of *Xist* have been precisely addressed by following the X-chromosome–wide enrichment of these histone marks during XCI [83]. This study demonstrated that PRC1-dependent H2AK119Ub accumulation precedes H3K27me3 deposition and also showed that *Xist* RNA lacking the A repeat is capable of recruiting PRC1 and PRC2 activity to regions of the Xi without active transcription, whereas spreading of both H3K27me3 and H2AK119Ub into domains of active genes is impaired [83]. Thus, active transcription seems to prevent the efficient spreading of Polycomb along the Xi, suggesting that the reduced accumulation of Polycomb-dependent histone marks observed in the absence of SPEN is likely related to impaired gene silencing. Although SPEN has been proven to act as a key player of *Xist*-mediated silencing, the exact molecular mechanism by which it contributes to the establishment and/or maintenance of gene silencing remains elusive. For example, whether SPEN’s role in XCI is restricted to the SPOC domain or whether other domains may recruit additional factors to the Xi is not yet clear nor is the impact of SPEN on the function of *Xist* in vivo. Indeed, SPEN knockout mice do not survive through embryonic development but reach E12.5, a time point that is never reached by female mice carrying *Xist* deletions, which fail to undergo XCI [46,87]. However, a knockout model to deplete the maternal pool of SPEN that would presumably enable early initiation of XCI in preimplantation embryos has never been performed.

### RBM15 and WTAP

Another *Xist* RNA interactor that was consistently identified is RNA-binding motif protein 15 (RBM15), a component of the N^6^-adenosine (m^6^A) RNA methylation machinery [73–75]. Interestingly, RBM15 interacts with Wilms tumor 1-associated protein (WTAP) [88], another subunit of the m^6^A methyltransferase complex, which was found to interact with *Xist* in an A-repeat–dependent manner [73]. m^6^A is the most abundant mRNA modification, and its impact on post-transcriptional gene regulation is being increasingly recognized [89–91]. Once established, this modification is recognized by a set of “m^6^A-readers”, such as the YT521-B homology (YTH) domain-containing proteins, which specifically binds to m^6^A and trigger downstream alterations affecting several aspects of mRNA metabolism, including alternative pre-mRNA splicing, 3’-end processing, nuclear export, translation, and decay [89]. The identification of RMB15 and WTAP as *Xist* RNA interactors pointed to a potential role of the m^6^A RNA methylation pathways in mediating the function of *Xist*. Indeed, human *XIST* RNA is methylated at 78 m^6^A residues, some of which encompass the repeat A element, and these modifications appeared to be required for proper gene silencing, although only a few loci along the Xi were tested [92]. Furthermore, *XIST* RNA methylation was reported to rely not only on the activity of RBM15 but also on its paralog RBM15B [92], also identified as a *Xist* interactor [75]. Both proteins are able to recruit the catalytic subunit of the (m^6^A) RNA methylation machinery METTL3 to *Xist* RNA in a WTAP-dependent manner, and their functional redundancy is likely to explain previously contradictory results obtained upon RBM15 depletion [74,78,92]. Indeed, RBM15 knockdown alone did not affect *Xist*-mediated silencing of the *Gpc4* gene as assessed by single molecule RNA FISH [74], and chromosome-wide analysis of X-linked gene activity revealed only a minor reduction in silencing efficiency in RBM15 knockout cells [78]. Importantly, double knockout of RBM15 and RBM15B seems to be essential for ESC viability which has meant that the exact role of these factors in XCI cannot be fully assessed [78]. Nevertheless, this study showed that deleting specific m^6^A sites in the 5’ region of *Xist* RNA results in a limited silencing defect, similarly to RBM15 and WTAP knockouts, indicating that the m^6^A pathway might not play a central role in *Xist-*mediated silencing, and highlighting the need to further assess the exact impact of m^6^A methylation on XCI [78]. For example, one of the open questions concerns the recognition of m^6^A residues along *Xist* by the YTHDC1 reader. Depletion of YTHDC1 was shown to result in defective XCI, whereas tethering YTHDC1 to *Xist* rescues the phenotype in the absence of a functional m^6^A methylation complex [92]. How YTHDC1 would execute this function and contribute to gene silencing remains to be defined. Another unexplored possibility is that m^6^A methylation recruited via *Xist* RNA to the X chromosome might affect the stability of X-linked mRNAs. In fact, although the exact impact of m^6^A on gene regulation is not yet fully understood, specific functions have been reported for at least some readers, resulting in opposite effects on gene expression. For example, binding of YTHDF1 improves the efficiency of translation of m^6^A-methylated mRNAs [93], whereas the YTHDF2 reader destabilizes its target mRNAs and promotes their degradation [94]. This different impact on mRNA stability might work as an effective strategy to potentially regulate XCI at a post-transcriptional level, leading to destabilization of mRNAs of the majority of X-linked gene, as well as increased stability of mRNAs transcribed from escaping genes that remain active along the otherwise silent Xi.

### hnRNP K

The heterogeneous nuclear ribonucleoprotein K (hnRNP K) was identified as an interactor of *Xist* by two proteomic approaches, although not in an A-repeat dependent manner [73–75]. However, none of the genetic screens confirmed its potential role as a key factor for *Xist*-mediated silencing [76,77]. Chu and colleagues initially showed that hnRNP K is recruited by *Xist* RNA independently of the A-repeat domain, and more recent studies have shown this interaction to be dependent on *Xist* RNA repeats B and C [62,63]. Functionally, Chu and colleagues validated the impact of hnRNP K on *Xist*-mediated silencing by knockdown experiments and were able to show that its depletion significantly reduces the accumulation of H3K27me3 and H2AK119ub along Xi [73]. Subsequently, hnRNP K was shown to recruit PRC1 to the Xi by binding the noncanonical PCGF3/5-PRC1 complex [63]. In particular, a 600-bp element encompassing *Xist* repeat B and a small part of repeat C (i.e., the *Xist* RNA Polycomb Interaction Domain, XR-PID) was identified as the required element to mediate hnRNP K/*Xist* interaction, and deletions of XR-PID abolished Polycomb recruitment and resulted in reduced gene silencing [63]. However, as this strong silencing defect was observed upon deletion of the XR-PID element but in the presence of a functional repeat A, this observation is difficult to interpret. One possible explanation is the nature of the *Xist* transgene itself, as this study employed a short form of *Xist* RNA encompassing repeats A, F, B, and part of C, but lacking the elements downstream the first 3.9 kb of *Xist* exon 1. Indeed, the lack of these sequences might somehow enhance the role of Polycomb recruitment in silencing establishment. More recently, three independent studies further explored the contribution of Polycomb recruitment to the establishment and maintenance of XCI by generating a series of *Xist* mutants in which repeats B and C have been deleted from the *Xist* endogenous locus [60,62,78]. The effect on gene silencing along the Xi varies in these studies, most likely reflecting the different time points at which X-linked gene expression was assessed during XCI. Nevertheless, none of the studies reported complete abrogation of XCI initiation. Thus, as gene silencing can be established upon induction of a defective *Xist* RNA that lacks repeats B and C and is unable to recruit PRC1/PRC2, although slightly less efficiently, Polycomb recruitment and transcriptional silencing appear to be largely uncoupled [62] Accordingly, the hnRNP K–dependent accumulation of H3K27me3 and H2AK119ub along the Xi seems to be necessary to stabilize silencing during XCI, rather than initiating it [62]. This model is in line with the observation that the subset of X-linked genes that are not silenced upon deletion of HDAC3 don’t accumulate Polycomb marks upon *Xist* RNA spreading [83]. Regardless of the interplay between Polycomb recruitment and gene silencing establishment, the initial recruitment of noncanonical PRC1 activity to the Xi leads to PRC2 accumulation, indicating that PRC1 is responsible for the *Xist*-dependent recruitment of PRC2 to the Xi [64]. Accordingly, deleting the core catalytic subunit of PRC1, RING1A/B, or the non-canonical PRC1 components PCGF3 and PCGF5 strongly reduced the *Xist*-dependent deposition of H2AK119ub and H3K27me3 during XCI [64]. The recruitment of PRC2 downstream of PRC1 along the Xi is supported by several other lines of evidence, including the fact that none of the core PRC2 components were identified in the proteomic studies, the identification of PRC1 components as *Xist* interactors [73], and the relative dynamics of H2AK119ub accumulation along the Xi early during XCI, occurring upstream of H3K27me3 [83].

### LBR

Another factor that has been proposed to associate with *Xist* RNA is the Lamin B receptor (LBR). LBR is a transmembrane protein anchored to the inner nuclear membrane where it binds Lamin B and works as a structural scaffold for proteins involved in chromatin silencing at the nuclear lamina [95]. McHugh and colleagues initially showed impaired silencing of two X-linked genes upon LBR knockdown [74]. Subsequently, Chen and colleagues identified the LBR element required to mediate the interaction with *Xist* RNA and also showed that losing this interaction results in defective gene silencing [96]. The same study proposed LBR to play a key role in recruiting the Xi to the nuclear lamina, suggesting that this nuclear repositioning might be required for XCI [96]. However, the *Xist*-coated chromosome can still be recruited to the nuclear lamina in case of a major silencing defect, for example, upon SPEN depletion [96], and the actively transcribed Xa has been shown to localize to the nuclear lamina in both male and female nuclei [97], indicating that the recruitment of the Xi to the nuclear lamina is not sufficient to initiate gene silencing. Furthermore, LBR mutant mice do not show a clear sex bias in embryonic lethality as would be expected in case of impaired XCI [98,99]. More recently, Nesterova and colleagues performed an X-chromosome–wide analysis of *Xist*-mediated silencing upon differentiation of LBR knockout ESCs and were able to show that lack of LBR leads to a minor silencing defect [78]. Deleting the element of *Xist* RNA mediating the interaction with LBR resulted in slightly more affected gene silencing, but the overall effect on XCI remained weak. Thus, although the LBR–*Xist* interaction might be necessary to maintain and/or stabilize gene repression, the exact role of LBR during XCI still remains unclear.

## Interactors of *Xist* implicated in *Xist* RNA chromatin association

One of the most fascinating and yet open questions about the function of *Xist* concerns how it remains associated only with the chromosome *in cis* rather than diffusing to neighboring chromosomes *in trans*. Based on RNA fluorescent in situ hybridization analysis (FISH), the *Xist* RNA domain appears to be confined to the same nuclear territory of Xi throughout the entire cell cycle [100], although human *XIST* was reported to dissociate from the Xi during mitosis [2,101], and this observation was confirmed by live-cell imaging in mouse cells [102]. Regardless of the fact that *Xist* RNA might be temporarily displaced from the Xi through cell division, its localization does not seem to rely on a sequence-specific mechanism. For example, neither RNase H nor DNase treatment affects the *Xist* RNA domain within the nuclear space, indicating that *Xist* does not bind the Xi simply via formation of RNA/DNA hybrids [101]. Rather, as *Xist* RNA remains with the nuclear matrix fraction after removal of chromosomal DNA, its localization to the Xi chromatin might be directed by proteins of the nuclear matrix [101].

### hnRNP U (SAF-A)

The matrix attachment protein hnRNP U has been proposed to play a key role in *Xist* RNA localization to chromatin. hnRNP U is enriched along the Xi [103,104], and its interaction with *Xist* was confirmed in proteomic studies [73–75]. In particular, hnRNP U seems to directly interact with exons 1 and 7 of both human and mouse *XIST*/*Xist* RNA [56,105]. Several knockdown studies validated the role of hnRNP U on *Xist* localization by reporting diffusion of *Xist* RNA from the Xi territory upon hnRNP U depletion [61,73,74]. Nevertheless, hnRNP U is dispensable for the localization of human *XIST* [106], and its requirement to localize *Xist* RNA seems to be at least partially cell type specific, indicating that *Xist* localization is likely to rely on a combination of anchoring factors rather than on hnRNP U alone [106,107]. Furthermore, the impact of hnRNP U on XCI establishment and/or maintenance remains to be fully understood. Indeed, hnRNP U was initially suggested to be necessary for the establishment of gene silencing [61], but given its recruitment to the Xi at a late time point during XCI, its functional relevance is more likely related to XCI maintenance [104].

### CIZ1

Cip1-interacting zinc finger protein 1 (CIZ1) is another nuclear matrix protein identified as a *Xist* interactor [73] and recently found to contribute to *Xist* localization [57,58]. CIZ1 has been originally characterized as a binding factor of key regulators of DNA replication and contrary to hnRNP U, is recruited by *Xist* RNA during the earliest stages of XCI [57,58]. Functionally, it comprises several domains, including three DNA binding zinc finger motifs [108] and a C-terminal nuclear matrix–anchoring MH3 domain (matrin 3-homologous domain 3) [109]. Thus, one tempting hypothesis is that CIZ1 might act as a bifunctional protein scaffold able to bind the Xi DNA via its zinc finger motifs and *Xist* RNA via the C-terminal nuclear matrix–anchoring domain. Indeed, the C-terminal domain is required to recruit CIZ1 to Xi, and deleting the E repeat within exon 7 of *Xist* abolishes this recruitment [58]. Nevertheless, whether CIZ1 directly interacts with *Xist* RNA or rather anchors the Xi via binding to other *Xist* RNA interactors is not yet clear. It should be noted that in hnRNP U knockout cells, CIZ1 remains localized with *Xist* RNA, although *Xist* RNA is dispersed throughout the nucleoplasm. Thus, CIZ1 and hnRNP U interact with *Xist* independently of each other, highlighting the complexity of the mechanisms directing *Xist* RNA coating and the formation of the Xi territory [57]. Finally, CIZ1 depletion results in dispersed *Xist* localization in mouse embryonic fibroblast (MEFs) and activated B and T lymphocytes but is certainly dispensable for XCI initiation as knockout mice survive embryonic development [58]. Nevertheless, CIZ1 null mice develop 100% penetrant female-specific lymphoproliferative disorder, indicating that loss of proper *Xist* localization in lymphocytes and impaired XCI might be the cause of the sex-specific phenotype. Consistent with this, deletion of *Xist* RNA in the mouse blood compartment was previously shown to lead to hematologic cancer [110]. However, none of these studies precisely assessed the allele-specific reactivation of X-linked genes along the Xi, so that the interplay between *Xist* RNA delocalization, X-linked gene dosage, and cancer development is yet to be clearly defined.

## Factors involved in chromosome-wide 3D structural reorganization of the Xi

Another remarkable feature of *Xist* RNA is its ability to direct a major reorganization of the 3D spatial architecture of the Xi. Evidence of such structural reshaping was originally provided by assessing the local organization of X-linked genes during XCI [86]. In particular, this study revealed that upon gene silencing X-inactivated genes are relocalised from the periphery of the *Xist* RNA domain to a more internal compartment, which is depleted of RNA Pol II and general transcription factors, whereas genes that escape XCI remain external of the silent compartment [86]. Similarly, X-linked transcribed long interspersed nuclear elements (LINE) are spatially separated from the *Xist* RNA domain early upon XCI and only start to intermingle with it at a later stage, when silencing has occurred [111]. More recently, our understanding of the Xi’s structural changes occurring during XCI has been greatly enhanced by the development of the Chromosome Conformation Capture (3C) technologies, which measure the physical arrangement of DNA in the 3D nuclear space. 3C methods unveiled several levels of higher order chromatin folding, including sub-megabase–scale topologically associating domains (TAD), defined as regions of DNA that preferentially contact each other along the chromosomes [40,112]. Comparative Hi-C analysis of the Xi 3D structure in mouse, human, and macaque has revealed that in contrast to the Xa and autosomes, the Xi is relatively depleted of TADs and appear to be folded into an unusual bipartite structure in which two large megadomains are separated by a boundary region encompassing the DXZ4/Dxz4 macrosatellite [75,113–116]. However, how *Xist* RNA coating results in partitioning of the Xi into megadomains via DXZ4 is not yet clear. Similarly, the exact causality and temporal dynamics between the collapse of TADs along the Xi and the establishment of gene silencing also remains to be defined. For example, *Xist* RNA was proposed to direct the 3D organization of Xi by repelling architectural factors, thus possibly avoiding the establishment of a transcription-favorable chromatin status (Minajigi 2015). However, how *Xist* RNA can repulse a subset of factors involved in chromatin 3D organization remains elusive, as do the sequence elements within *Xist* RNA that would account for this function.

### SmcHD1

Recently, the structural maintenance of chromosomes flexible hinge domain containing 1 (SmcHD1) protein was hypothesized to participate in reshaping the 3D conformation of Xi [117–119]. SmcHD1 interacts with *Xist* RNA [75] and was previously found to be required for XCI maintenance, as its deletion in vivo does not affect XCI initiation but results in reactivation of approximately 10% of X-linked genes along Xi [120,121]. Mechanistically, SmcHD1 has previously been shown to allow for CpG island methylation along the Xi; however, this does not account for its function in maintaining X-linked gene silencing [121]. As SmcHD1 carries an SMC hinge domain normally found in proteins involved in chromosome condensation and compaction, its role in XCI maintenance might rather be structural. Indeed, knockdown of SmcHD1 leads to decompaction of the human Xi, similar to *XIST* depletion [122], and strengthening of TAD-like structures along Xi in several cell types [117–119], although whether this structural reorganization is accompanied by transcriptional changes remains unclear [117,119].

## Polycomb factors and Xi 3D organisation

Another unexplored possibility is that the distribution of chromatin features along the X chromosome before XCI initiation might direct its structural reorganization. For example, the involvement of the Polycomb complexes in directing the 3D folding of chromosomes is increasingly recognized [123–125]. In particular, genomic loci that are repressed by PRC1 have been shown to form self-interacting domains of compacted chromatin that are thought to exclude the transcriptional machinery and to transmit the silent state of PRC1 targets through cell division [124]. In the context of XCI, X-linked regions that preferentially accumulate Polycomb-dependent histone marks have been reported to be pre-marked by Polycomb prior to *Xist* spreading [83], indicating that indeed the distribution of Polycomb along the X before XCI might direct its spatial reorganization during XCI. In line with this hypothesis, the X-linked regions pre-marked by Polycomb are spatially located in close 3D proximity of the *Xist* locus and correspond to the regions that become first targeted by *Xist* RNA at the beginning of XCI [83,126].

Finally, Polycomb complexes might contribute to the 3D reorganization of the Xi by supporting the formation of membrane-less organelles similar to paraspeckles, defined as protein-rich nuclear condensates built around a specific lncRNA scaffold and able to influence gene regulation by sequestrating a subset of specific proteins by RNA-protein interactions (for review [127]). Indeed, PRC1-bound chromatin has been recently shown to undergo liquid–liquid phase separation (LLPS) to form nuclear aggregates that colocalize with H3K27me3-dense chromatin regions [128]. Interestingly, the potential involvement of phase-separated aggregates in shaping the 3D structure of Xi is not limited to PRC1 but is likely to concern other interactors of *Xist* RNA. For example, FUS, hnRNPA2B1, and RBM14 all carry low-complexity domains that are prone to mediate phase separation and were previously reported to be implicated in the formation of paraspeckles [127,129–131]. Given that phase-separated aggregates have been proposed to play a role in the formation of heterochromatin [132,133], an exciting hypothesis is that *Xist* RNA might direct the reorganization of Xi by forcing a local high concentration of specific proteins via RNA–protein interactions, thus creating a phase-separated silent compartment in which factors important for the establishment of facultative heterochromatin are sequestered. However, whether or not such a structure is assembled and its potential impact on the initiation and/or maintenance of XCI remains to be proven.

## Conclusions

Almost thirty years after its discovery, *Xist* RNA continues to provide a powerful model system for exploring a multitude of epigenetic mechanisms, including the developmentally regulated formation of facultative heterochromatin and the 3D organization of the genome in nuclear space. Future studies are likely to unveil the molecular mechanisms through which noncoding RNAs induce or change the 3D structure of the genome, as well as whether these changes in structure are a cause or a consequence of changes in gene activity and how they play a role in maintaining differences between active and inactive compartments of the mammalian genomes.
